# Ventilatory efficiency slope is associated with cardiopulmonary complications after thoracoscopic anatomical lung resection

**DOI:** 10.1093/icvts/ivac039

**Published:** 2022-02-14

**Authors:** Benoît Bédat, Evangelos Koliakos, Marco S Demarchi, Jean Perentes, Marc-Joseph Licker, Frédéric Triponez, Thorsten Krueger, Wolfram Karenovics, Michel Gonzalez

**Affiliations:** 1 Service of Thoracic Surgery, University Hospital of Lausanne, Lausanne, Switzerland; 2 Division of Thoracic and Endocrine Surgery, Geneva University Hospitals and University of Geneva, Geneva, Switzerland; 3 Division of Anesthesiology, Geneva University Hospitals and University of Geneva, Geneva, Switzerland

**Keywords:** Minute ventilation-to-carbon dioxide output slope, Ventilatory efficiency, Anatomical lung resection, Video-assisted thoracic surgery, Postoperative complications, Lung cancer

## Abstract

**OBJECTIVES:**

The aim of this study was to identify whether steeper V.E/V. CO_2_ slope was associated with cardiopulmonary complications (CPC) after anatomical resection by video-assisted thoracic surgery. Long-term survival was analysed as secondary outcome.

**METHODS:**

We reviewed the files of all consecutive patients who underwent pulmonary anatomical resections by video-assisted thoracic surgery between January 2010 and October 2020 at the Centre for Thoracic Surgery of Western Switzerland. Logistic regression was used to investigate the risk of CPC associated with the V.E/V.CO_2_ slope and other possible confounders. Survival was analysed with Kaplan–Meier curves. Risk factors associated with survival were analysed with a Cox proportional hazards model.

**RESULTS:**

The V.E/V.CO_2_ slope data were available for 145 patients [F/M: 66/79; mean age (standard deviation): 65.8 (8.9)], which were included in the analysis. Patients underwent anatomical resection [lobectomy (71%) or segmentectomy (29%)] mainly for lung cancer (96%). CPC and all-cause 90-day mortality were 29% and 1%, respectively. The mean (standard deviation) percentage of the predicted $\dot{\text{V}}$O_2peak_ was 70% (17). Maximum effort during cardiopulmonary exercise test was reached in only 31% of patients. The V.E/V.CO_2_ slope (standard deviation) was not different if the maximum effort was reached or not [39 (6) vs 37 (7), *P* = 0.21]. V.E/V.CO_2_ slope >35 was associated with an increased risk of CPC (odds ratio 2.9, 95% confidence interval 1.2, 7.2, *P* = 0.020). V.E/V.CO_2_ slope >35 was not associated with shorter survival censored for lung cancer-related death.

**CONCLUSIONS:**

$\dot{\text{V}}$ E/$\dot{\text{V}}$CO_2_ slope >35 is significantly associated with postoperative CPC after anatomical resections by video-assisted thoracic surgery.

**Clinical registration number CER-VD (Switzerland):**

Project ID: 2021-00620.

**Figure:**
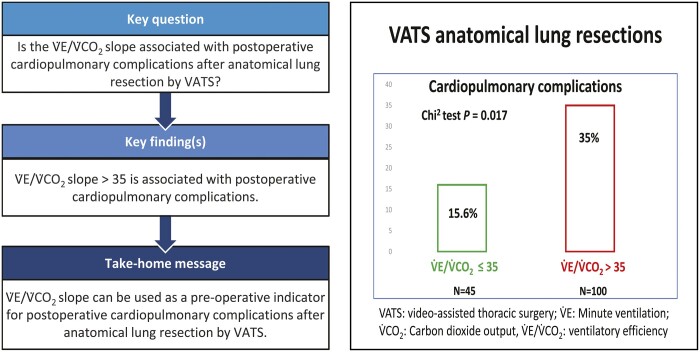

## INTRODUCTION

Pulmonary anatomical resection can be proposed for patients with early-stage non-small cell lung cancer, for benign lesions or for metastases. A video-assisted thoracic surgery (VATS) approach is now preferred because it decreases postoperative pain and improves quality of life as compared to a thoracotomy approach [1, 2]. To assess perioperative risks, lung function and low technology fitness tests are recommended as first-line measurements [3, 4]. A cardiopulmonary exercise test (CPET) is recommended as second-line assessment in high-risk patients to measure maximal oxygen consumption (V.O_2 max_) [5]. However, a low V.O_2 max_ does not seem to be a reliable predictor of increased surgical risk after VATS lobectomy according to recently published evidence [6]. Furthermore, some patients are unable to perform a maximum effort test due to leg fatigue, heart diseases, comorbidities or lack of motivation. In such cases, V.O_2 max_ can be replaced by the peak V.O_2_ at volitional incremental exercise [7].

The minute ventilation-to-carbon dioxide output (V.E/V.CO_2_) slope measured during CPET reflects the ventilatory efficiency and is gaining interest for thoracic surgery purposes. Previous studies showed that ventilatory inefficiency could predict postoperative complications, 90-day mortality and 2-year survival after pulmonary anatomical resections [8–11]. However, these studies included patients undergoing thoracotomy or pneumonectomy and authors chose various cut-off values of the V.E/V. CO_2_ slope.

We sought to identify whether V.E/V.CO_2_ slope correlates to cardiopulmonary complications (CPC) as primary outcome and long-term survival as secondary outcome after anatomical resection by VATS. We hypothesized that a higher V.E/V.CO_2_ slope value is associated with a higher rate of CPC and shorter survival.

## PATIENTS AND METHODS

### Ethics statement

The local ethics committee (CER-VD in Lausanne) approved this study on 30 March 2021 (referral number: 2021-00620) and waived the need to obtain informed patient consent due to the studied oncological pathology.

### Patients

We reviewed the records of all patients who underwent lobectomy or segmentectomy by VATS from January 2010 to December 2020 at the University Hospitals of Lausanne and Geneva in Switzerland. All surgical indications were included. Patients who underwent pneumonectomy and patients without V.E/V.CO_2_ slope data were excluded from the analysis. Patient records were extracted from the hospital data management system. The following data were obtained: patient demographics and age-adjusted Charlson comorbidity index (CCI); cardiac and pulmonary assessment; surgical indication; type of pulmonary resection; histological findings; length of hospital stay; all-cause 90-day mortality; and duration of long-term survival.

### Surgical technique

Four surgeons (M.G., W.K., T.K. and J.P.) carried out all anatomical resections by VATS included in this study. Surgical resections were undertaken using an anterior single to 3-port approach. All vascular structures were transected using endoscopic staplers or an energy device. Complete dissection of lymph nodes was carried out in patients with non-small cell lung cancer. All bronchial structures were transected using endoscopic staplers. For segmentectomies, the intersegmental plane was divided using staplers. Segmentectomies are classified as simple (culminectomy, lingulectomy, apical or basilar segments) or complex (individual or bi-segmentectomy). For lobectomies, a fissure-less technique was preferred. Postoperative treatment focused on pain control by opioid drugs, early mobilization and chest physiotherapy.

### Pulmonary assessment

All patients completed a preoperative symptom-limited CPET preceded by pulmonary function tests. The forced expiratory volume in 1 s and the Diffusing Capacity of the Lung for carbon monoxide (DLCO) were also expressed as percentages of the predicted values for age, gender and height. The predicted postoperative functions were calculated according to the number of resected segments [3]. The CPET was performed in case of low lung function, on an upright, electronically braked cycle ergometer with breath-by-breath expired gas analysis. The test was stopped if major dyspnoea or if significant changes appeared on the electrocardiogram (ECG) or in the blood pressure. V.O_2 peak_ was determined as the highest average value over 20 s [12]. The V.E/V.CO_2_ slope was calculated by linear regression analysis from start of exercise to anaerobic threshold. Maximal effort was determined based on respiratory exchange ratio >1.10 and maximal heart rate >85% of age-adjusted predicted maximal heart rate.

### Postoperative complications

The following CPC were chosen for analysis: atrial fibrillation; acute myocardial ischaemia; heart failure; pneumothorax; prolonged air leak, defined as an air leak lasting beyond postoperative Day 7; acute respiratory distress syndrome, defined using the Berlin classification [13]; pneumonia, defined by the need for antibiotics following appearance of new lung infiltrate on chest-X rays, fever, or an elevated white blood cell count > 12 000 per ml; atelectasis; and pulmonary embolism (confirmed by V/Q scan or computed tomography scan).

### Statistical analysis

Continuous variables following a normal distribution are presented as means with standard deviation. Nominal variables with ordered categories are summarized as medians with interquartile range. Binary variables are presented as numbers with percentages. A chi-squared or Fisher’s exact test was used to analyse categorical variables. A *T*-test or Mann–Whitney *U*-test was used to compare continuous variables. We compared the occurrence of CPC with the V.E/V.CO_2_ slope. V.E/V.CO_2_ slope cut-offs >35 and >40 were used, which, as previously described, were associated with increased postoperative complications and mortality [9, 10, 14]. A *P*-value <0.05 was considered statistically significant.

Univariable logistic regression was initially used to screen variables associated with CPC. The following variables were tested: age, sex, body mass index, CCI, smoking status, forced expiratory volume in 1 second (%), DLCO (%), predicted V.O_2peak_ (%), V.E/V.CO_2_ slope, workload (Watts), induction chemotherapy, type of operation and pTNM status. Log-linearity assumptions were tested. Multivariable analysis was not performed because only the V.E/V.CO_2_ slope was found to be significant in the univariable analysis.

Time-to-event analysis was done using death censored for lung cancer-related death as the event. Patients without malignancy and with carcinoid tumour have been excluded for the survival analysis. Patients were censored at the time of their last follow-up visit. Kaplan–Meier estimates were assessed for the 2 V.E/V.CO_2_ slope groups (≤35 and >35). Log-rank tests were used to compare differences in Kaplan–Meier estimates. Cox proportional hazards regression was used to investigate the association between the survival and V.E/V.CO_2_ slope as well as possible confounders as independent variables (age, CPC and pTNM status). All analyses were performed using STATA software, version 14 (StataCorp LLC, TX, USA).

## RESULTS

**Figure 1:**
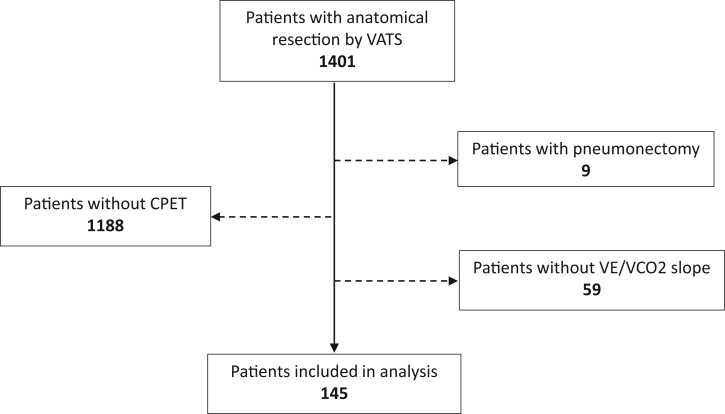
Flow diagram for study participants. CPET: cardiopulmonary exercise test; VATS: video-assisted thoracic surgery; V.E: Minute ventilation (l/min); V.CO_2_: Carbon dioxide output (l/min).

In total, 1401 patients underwent anatomical resection by VATS in the 2 hospitals during the study period. CPET was performed in 204 patients (15%). Patients who had CPET had lower lung function (Table 1). The $\dot{\text{V}}$E/$\dot{\text{V}}$CO_2_ slope data, available for 145 patients [F/M: 66/79; mean age (standard deviation): 65.8 (8.9)], were included in the analysis (Fig. 1). Patients underwent anatomical resection [lobectomy (71%) or segmentectomy (29%)] mainly for lung cancer (96%; Table 2). Conversion rate was 4%, the causes being 3 haemorrhages, 2 pleural adhesions and 1 technical difficulty. The CPC rate and 90-day mortality were 29% and 1%, respectively.

**Table 1:** Patient characteristics according to cardiopulmonary exercise test performance

Variables	Without CPET	With CPET and $\dot{\text{V}}$E/$\dot{\text{V}}$CO_2_ slope	*P*-value
	*N* = 1197	*N* = 145	
Age, mean (SD)	65.6 (0.3)	65.8 (8.9)	0.89
Gender, female (%)	565 (47)	66 (45)	0.70
BMI, mean (SD)	25.2 (4.9)	24.1 (5)	0.011
Pack-year, mean (SD)	28.8 (30.4)	40.8 (29.9)	<0.001
CCI, median [IQR]	2 [0–4]	2 [1–4]	0.91
FEV1 % predicted, mean (SD)	88.7 (21.4)	75.8 (18.6)	<0.001
DLCO % predicted, mean (SD)	76.5 (20.2)	59.8 (16.3)	<0.001
Ejection fraction %, mean (SD)	63 (7)	62.9 (8)	0.83
Lung cancer (%)	979 (82)	139 (96)	<0.001
pT staging >pT1 (%)	425 (43)	80 (57)	0.002
pN0 (%)	787 (81)	111 (80)	0.77
Segmentectomy (%)	458 (38)	42 (29)	0.029
Cardiopulmonary complications (%)	317 (26)	42 (29)	0.52
Drainage duration, median day [IQR]	2 [1–5]	3 [2–6]	0.054
LOS, median day [IQR]	6 [4–10]	8 [6–12]	<0.001

CCI: Charlson comorbidity index; DLCO: Diffusing capacity of the lung for carbon monoxide; FEV1: forced expiratory volume in one second; IQR: interquartile range; LOS: length of hospital stay; SD: standard deviation.

**Table 2:** Patient characteristics according to the V.E/V.CO_2_ slope with a cut-off of 35

Variables	Overall	$\dot{\text{V}}$ E/$\dot{\text{V}}$CO_2_ ≤35	$\dot{\text{V}}$ E/$\dot{\text{V}}$CO_2_ >35	*P*-value
	*N* = 145	*N* = 45	*N* = 100	
Age, mean (SD)	65.8 (8.9)	63.5 (9.9)	66.8 (8.3)	0.036
Gender, female (%)	66 (45)	27 (60)	39 (39)	0.019
BMI, mean (SD)	24.1 (5)	23.8 (4.7)	24.3 (5.1)	0.57
Hypertension (%)	70 (48)	22 (49)	48 (48)	0.92
Atrial fibrillation (%)	20 (14)	4 (9)	16 (16)	0.25
Diabetes (%)	17 (12)	2 (4)	15 (15)	0.068
Heart failure (%)	4 (3)	1 (2)	3 (3)	1.0
History of myocardial infarction (%)	12 (8)	2 (4)	10 (10)	0.34
Tobacco (%)				0.34
No smoker	7 (5)	4 (9)	3 (3)	
Active	92 (63)	27 (60)	65 (65)	
Former	46 (32)	14 (31)	32 (32)	
Pack-year, mean (SD)	40.8 (29.7)	33.2 (27)	44.2 (30.4)	0.038
CCI, median [IQR]	2 [1-4]	1 [0-2]	2.5 [1-5]	<0.001
FEV1 % predicted, mean (SD)	75.8 (18.6)	78.9 (21.1)	74.5 (17.3)	0.19
DLCO % predicted, mean (SD)	59.8 (16.3)	66.8 (18.6)	56.5 (14.1)	<0.001
Ppo FEV1 %, mean (SD)	62.3 (15.1)	63.2 (17.4)	61.9 (14)	0.62
Ppo DLCO %, mean (SD)	49.1 (13.3)	53.2 (13.9)	47.3 (12.7)	0.013
Ejection fraction %, mean (SD)	62.9 (7.7)	62.2 (6.4)	63.2 (8.3)	0.53
V.O_2 peak_, mean (SD)	16.8 (4.1)	18.3 (4.5)	16.2 (3.7)	0.003
V.O_2 peak_ predicted %, mean (SD)	70 (16.7)	77.2 (16)	67 (16.2)	0.001
Ppo V.O_2 peak_ predicted, mean (SD)	57.8 (14.6)	62.5 (14.8)	55.9 (14.2)	0.015
Workload, Watts, mean (SD)	82.3 (25.2)	91.5 (28.6)	78.2 (22.4)	0.003
RER, mean (SD)	1.11 (0.13)	1.15 (0.15)	1.1 (0.12)	0.026
Heart rate % predicted, mean (SD)	83.5 (13.9)	84.5 (10.9)	83.1 (15.1)	0.59
Indication for surgery (%)				0.17
Lung cancer	139 (96)	45 (100)	94 (94)	
Other	6 (4)	0	6 (6)	
Neoadjuvant chemotherapy (%)	6 (4)	0	6 (6)	NA
Histology of lung cancer (%)				0.95
NSCLC	132 (95)	43 (96)	89 (95)	
Carcinoid	3 (2)	1 (2)	2 (2)	
SCLC/LCNEC	4 (3)	1 (2)	3 (3)	
pT staging (%)				0.11
pT1	59 (42)	20 (44)	39 (41)	
pT2	56 (40)	22 (49)	34 (36)	
pT3	18 (13)	2 (4)	16 (17)	
pT4	6 (4)	1 (2)	5 (5)	
pN0 (%)	111 (80)	37 (82)	74 (79)	0.63
Type of resection by VATS (%)				0.11
Segmentectomy	42 (29)	9 (20)	33 (33)	
Simple	23 (55)	4 (44)	19 (58)	
Complex	19 (45)	5 (56)	14 (42)	
Lobectomy	103 (71)	36 (80)	67 (67)	
Sleeve lobectomy (%)	4 (3)	0	4 (4)	NA
Right upper lobe	2 (1)	0	2 (2)	
Right lower lobe	1 (1)	0	1 (1)	
Left lower lobe	1 (1)	0	1 (1)	
Conversion (%)	6 (4)	2 (4.4)	4 (4)	1.0
Cardiopulmonary complications (%)	42 (29)	7 (16)	35 (35)	0.017
Drainage duration, median day [IQR]	3 [2-6]	3 [2-5]	4 [2-6]	0.57
LOS, median day [IQR]	8 [6-12]	7 [6-10]	8 [6-13]	0.067
Death at 90 days (%)	1 (1)	0	1 (1)	NA
Follow-up median day [IQR]	630 [199-11291]	1046 [295-1894]	590 [132-971]	0.029

BMI: body mass index; CCI: Charlson comorbidity index; DLCO: diffusing capacity of the lung for carbon monoxide; FEV1: forced expiratory volume in one second; LNEC: large cell neuroendocrine carcinoma; LOS: length of hospital stay; NSCLC: non-small cell lung cancer; Ppo FEV1: predicted postoperative FEV1; RER: respiratory exchange ratio; SCLC: small-cell lung cancer; V.CO_2_: Carbon dioxide output (l/min); V.E: Minute ventilation (l/min).

### Cardiopulmonary exercise test

Most patients (*N* = 100, 69%) had a V.E/V.CO_2_ slope >35 (Table 2). Maximum effort during CPET was reached by only 31% of patients. The mean (standard deviation) V.E/V.CO_2_ slope did not differ if the maximal effort was reached or not [36.6 (5.4) vs 38.7 (7.3), respectively, *P* = 0.053]. Patient characteristics according to the V.E/V.CO2 slope are summarized in Table 2. Older patients, high CCI and high pack-year number were associated with poor ventilatory efficiency (V.E/V.CO_2_ slope > 35), as well as lower DLCO, V.O_2peak_, workload and respiratory exchange ratio. Predicted V.O_2peak_ was between 35% and 75% in 65% of patients and >75% in 35% of patients.

### Cardiopulmonary complications

Patients who underwent a CPET did not have more CCP (Table 1). The types of CPC are not associated with the V.E/V.CO_2_ slope (Supplementary Material, Table S1). In the univariable analysis (Table 3), a V.E/V.CO_2_ slope >35 was associated with an increased risk of CPC (odds ratio 2.9, *P* = 0.020). We can see that the risk of CPC tended then to increase with the steepness of the slope with a higher risk in patients with a V.E/V.CO_2_ slope >40 (Table 3). Amongst patients with predicted V.O_2peak_ >75%, the rate of CPC was higher if the $\dot{\text{V}}$E/$\dot{\text{V}}$CO2 slope was >35 (41% vs 5% if slope ≤35, *P* = 0.004). The predicted V.O_2peak_ did not affect the occurrence of complications whether the maximum effort was achieved or not (odds ratio 0.9, 95% confidence interval 0.8, 1.1, *P* = 0.34; odds ratio 1, 95% confidence interval 0.9, 1.1, *P* = 0.98, respectively).

**Table 3:** Univariable logistic regression model of risk factors for cardiopulmonary complications after pulmonary resection by VATS

Variables	Cardiopulmonary complications	
	Odds ratio (95% CI)	*P*-value
V.E/V.CO_2_ slope >35 (vs ≤35)	2.9 (1.2, 7.2)	0.020
V.E/V.CO_2_ slope >40 (vs ≤40)	1.8 (0.9, 3.8)	0.10
V.E/V.CO_2_ slope (ref ≤35)		
>35 - ≤40 (*N* = 46)	2.6 (0.9, 7.2)	0.06
>40 (*N* = 54)	3.2 (1.2, 8.5)	0.020
Age	1 (0.9, 1)	0.95
BMI	1 (0.9, 1)	0.23
Pack-year	1 (1, 1)	0.96
V.O_2 peak_ predicted	1 (0.9, 1)	0.16
Maximal effort reached (vs no reached)	0.8 (0.3, 1.8)	0.51
Workload	1 (1, 1)	0.092
DLCO % predicted	1 (1, 1)	0.77
FEV1 % predicted	1 (1, 1)	0.65
CCI	0.9 (0.8, 1.1)	0.37
Neoadjuvant chemotherapy	2.8 (0.6, 14.5)	0.22
pT1 (vs >pT1)	0.8 (0.4, 1.8)	0.59
pN+ (vs pN0)	0.5 (0.2, 1.6)	0.25
Lobectomy (vs segmentectomy)	1 (0.5, 2.3)	0.95

BMI: body mass index; CCI: Charlson comorbidity index; CI: confidence interval; DLCO: diffusing capacity of the lung for carbon monoxide; FEV1: forced expiratory volume in one second.

### Overall survival

**Figure 2:**
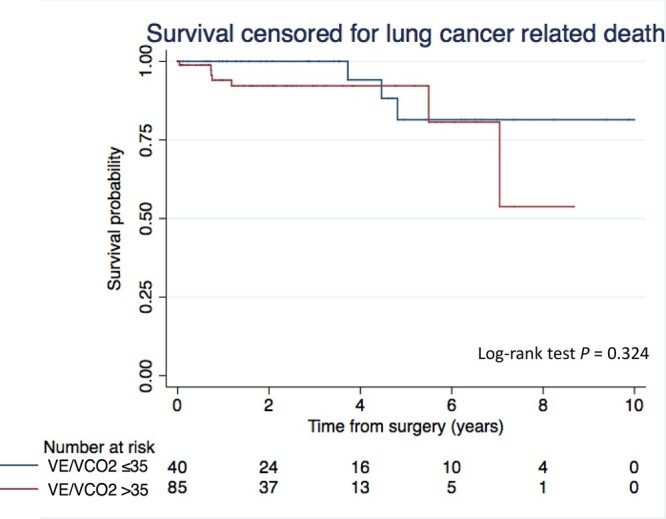
Kaplan–Meier curves of survival censored for lung cancer-related death after anatomical resection by VATS according to the V.E/V.CO_2_ slope. The log-rank test was used to compare differences in Kaplan–Meier estimates. VATS: video-assisted thoracic surgery; V.E: Minute ventilation (l/min); $\dot{\text{V}}$CO_2_: Carbon dioxide output (l/min).

The median follow-up was 630 days. Nineteen patients (13%) died during the follow-up period. The cause of death was lung cancer progression in 9 patients (47%) and underlying disease in 10 patients (53%). A $\dot{\text{V}}$E/$\dot{\text{V}}$CO_2_ slope >35 was not associated with lower survival censored for lung cancer-related death as compared to a V.E/V.CO_2_ slope ≤35 (Fig. 2). In the univariable analysis, V.E/V.CO_2_ slope >35, age, the presence of CPC and the pTNM status were not associated with a shorter survival (Table 4).

**Table 4:** Univariable Cox Proportional Hazards model for survival analysis censored for lung cancer-related death after pulmonary resection by VATS

Variables	Overall survival	
	Hazard ratio (95% CI)	*P*-value
$\dot{\text{V}}$ E/$\dot{\text{V}}$CO_2_ slope >35 (vs ≤35)	2 (0.5, 7.9)	0.33
$\dot{\text{V}}$ E/$\dot{\text{V}}$CO_2_ slope >40 (vs ≤40)	3 (0.8, 10.8)	0.09
Age	1 (1, 1.1)	0.27
CCI	1.1 (0.9, 1.3)	0.44
Cardiopulmonary complications	1.2 (0.3, 4.7)	0.78
>pT1 (vs pT1)	1.1 (0.2, 4.9)	0.91
pN+ (vs pN0)	2 (0.4, 10.3)	0.41

CCI: Charlson comorbidity index; CI: confidence interval; V.E: minute ventilation (l/min); V.CO_2_: carbon dioxide output (l/min).

## DISCUSSION

In this 2-centre retrospective cohort study, we found that V.E/V.CO_2_ slope >35 was a predictor of early CPC after anatomical pulmonary resection by VATS.

Risk assessment before anatomical pulmonary resection is recommended and widely used with functional measurements [3]. Amongst CPET results, V.O_2max_ is the most widely used variable with a minimal prohibitive threshold at 10 ml/kg/min or 35% of the predicted value [3]. However, it requires maximum effort to obtain the most reliable diagnostic. Other indicators resulting from the CPET, such as the V.E/V.CO_2_ slope, can be used for lung resection. The relationship between V.E and $\dot{\text{V}}$CO_2_ reflects the increase in ventilation in response to CO_2_ production, and thus shows the ventilatory efficiency. The V.E/V.CO_2_ slope can be measured at sub-maximal workload levels, as demonstrated in our study. A higher V.E/V.CO_2_ slope is classically observed in patients with heart failure, pulmonary embolism, pulmonary hypertension or restrictive lung disease and is associated with an increased risk of cardiac-related events and mortality [14–17].

Previous studies assessed the association of preoperative $\dot{\text{V}}$E/$\dot{\text{V}}$CO_2_ slope with outcomes after lung resection. Torchio *et al.* [8] showed that a V.E/V.CO_2_ slope >34 predicted mortality at 30 days in chronic obstructive pulmonary disease patients. However, all patients in their study underwent resections by thoracotomy (*N* = 145) including 27% who underwent a pneumonectomy. Brunelli *et al.* [9] reported increased postoperative respiratory complications in 225 patients (including 28 pneumonectomies, all by thoracotomies) when higher V.E/V.CO_2_ slope was higher (35 vs 31), making V.E/V.CO_2_ slope a better predictor of respiratory complications than peak V.O_2_. Similarly, Shafiek *et al.* [10] concluded that a V.E/V.CO_2_ slope >35 was associated with an increased risk of postoperative complications or mortality in 82 chronic obstructive pulmonary disease patients. Finally, Miyazaki *et al.* [11] reported that a V.E/V.CO_2_ slope >40 was associated with a higher 90-day mortality and a shorter overall survival at 2 years after lobectomy or segmentectomy by thoracotomy or by VATS.

In our study, we found that higher CCI and lower DLCO were associated with a $\dot{\text{V}}$E/$\dot{\text{V}}$CO_2_ slope >35. However, $\dot{\text{V}}$E/$\dot{\text{V}}$CO_2_ slope >35 was associated with CPC, regardless of the presence of moderate to severe chronic obstructive pulmonary disease or comorbidities, thus confirming results reported by Brunelli *et al.* [9]. The risk of CPC tended then to increase with the steepness of the slope, with a higher risk in patients with a $\dot{\text{V}}$E/$\dot{\text{V}}$CO_2_ slope >40. Our study did not establish any association between the V.E/V.CO_2_ slope and the occurrence of prolonged air leak or other particular complications, as mentioned in Brat *et al.* [18]. We showed that V.O_2peak_ is significantly reduced in patients with ventilatory insufficiency, suggesting reduced exercise capacity in these patients. Yet, V.O_2peak_ was not strongly associated with short- and long-term outcomes in our study, a finding that aligns with results reported by Begum *et al.* [6]. Interestingly, even in patients with higher levels of predicted V.O_2peak_, CPC occurs more frequently with a $\dot{\text{V}}$E/$\dot{\text{V}}$CO_2_ slope >35, as previously reported by Brunelli *et al.* [9]. This result confirms that the interpretation of these 2 variables should be differentiated, as has been proposed in patients with heart failure [19]. Contrary to other studies, we did not show an association between V.E/V.CO_2_ slope and long-term survival [11, 20]. However, we used a different cut-off and we censored death related to lung cancer.

The physiological determinants of V.E/V.CO_2_ slope are insufficiently known. For Miyazaki *et al.*, the presence of a latent subclinical heart failure and an increased postoperative ventilation-perfusion mismatch could explain an increased mortality after lung resection. This is in keeping with the fact that V.CO_2_ reflects alveolar perfusion, which is reduced in cases of decreased cardiac output. In addition, in patients with heart failure, a ventilatory drive increases resulting in a steeper V.E/V.CO_2_ slope. Bobbio *et al.* [21] showed that the V.E/V.CO_2_ slope increased significantly 3 months after lobectomy. That could be explained by well-documented postoperative right ventricular dysfunction after lobectomy, or by lung atelectasis [22]. This might also explain postoperative exercise limitations and exaggerated ventilatory drive. Furthermore, preliminary results on the effect of a short-term pre-habilitation programme on V.E/V.CO_2_ slope have reported either no effect [23, 24] or a slight improvement of the ventilator efficiency [25], but further larger studies are required to confirm these results.

Finally, current recommendations are based on studies that include patients who underwent lobectomy or greater resections by thoracotomy [2]. In lung resections by VATS, the use of $\dot{\text{V}}$O_2 max_ for perioperative risk assessment has been questioned [5]. Some studies evaluated the association of lung function and CPET results with perioperative risks after VATS anatomical resections compared to thoracotomy procedure, but the reported results are contradictory [26, 27]. New recommendations should be proposed according to current practices.

###  

This study has potential limitations. Firstly, the retrospective design can introduce a selection bias. The preoperative selection of patients did not depend on the V.E/V.CO_2_ slope value, hence minimizing this bias. The CPC were defined a priori and recorded prospectively. Secondly, a control group is lacking, introducing a sampling bias. Third, the small sample size could preclude certain statistical methods. For this reason, analysis of 90-day mortality was not possible and the results regarding survival and V.O_2peak_ should be interpreted cautiously, as should the lung function analysis. Fourth, the 2 centres included in this study used 2 different systems for CPET analysis, introducing a measurement bias. However, the test protocols were the same and the rate of complications did not differ between the centres, which shows that this bias would have been minimized. To resolve these limitations, a larger, possibly multicentre study would be needed.

In conclusion, we found that V.E/V.CO_2_ slope >35 is significantly associated with postoperative CPC. CPET and $\dot{\text{V}}$E/$\dot{\text{V}}$CO_2_ slope analysis should be investigated in a large cohort study to refine preoperative risk assessment, including for patients undergoing VATS procedures and segmentectomies, currently missing in general recommendations. Finally, particular caution should be exercised during the postoperative period in patients with $\dot{\text{V}}$E/$\dot{\text{V}}$CO_2_ slope >35 and early discharge should be considered carefully.

## Supplementary Material

ivac039_Supplementary_Data

## Notes

Presented at the 29th European Conference on General Thoracic Surgery, 20–22 June 2021 Virtual meeting.

## ABBREVIATIONS

CCI: Charlson comorbidity index
CPC: Cardiopulmonary complications
CPET: cardiopulmonary exercise test
DLCO: Diffusing Capacity of the Lung for carbon monoxide
$\dot{\text{V}}$ E: Minute ventilation
$\dot{\text{V}}$ CO_2_: Carbon dioxide output
VATS: Video-assisted thoracic surgery
