# Supplementary material for: Ventilatory efficiency slope is associated with cardiopulmonary complications after thoracoscopic anatomical lung resection
Source: Interact Cardiovasc Thorac Surg. 2022 Feb 14;35(1):ivac039. doi: 10.1093/icvts/ivac039 (PMC9252121; doi:10.1093/icvts/ivac039)
Supplement: ivac039_Supplementary_Data [file ivac039_supplementary_data.docx]

Supplementary Table 1. Type of cardiopulmonary complications according to the V̇E/V̇CO_2_ slope

| **Variables** | **V̇E/V̇CO_2_ ≤35**  **n=45** | **V̇E/V̇CO_2_ >35**  **N=100** | **P-value** |
| --- | --- | --- | --- |
| Bronchopneumonia (%) | 4 (8.9) | 23 (23) | 0.068 |
| Prolonged air leak (%) | 4 (8.1) | 17 (17) | 0.199 |
| Pneumothorax (%) | 0 | 8 (8) | 0.058 |
| ARDS (%) | 1 (2.2) | 1 (1) | 0.559 |
| Pulmonary embolism | 0 | 2 (2) | 1.000 |
| Atrial fibrillation (%) | 1 (2.2) | 8 (8) | 0.274 |
| Myocardial infarction (%) | 0 | 1 (1) | 1.000 |
| Heart failure (%) | 0 | 1 (1) | 1.000 |

ARDS: acute respiratory distress syndrome; V̇E: Minute ventilation (L/min); V̇CO_2_: Carbon dioxide output (L/min)
